# Safety of dupilumab in Chinese pediatric patients aged 6 months and older: a prospective real-world study

**DOI:** 10.3389/fped.2024.1524962

**Published:** 2025-01-17

**Authors:** Yanhua Chen, Jiang Ni, Ming Li, Yuan Hong, Kouzhu Zhu, Rong Hong, Li Deng, Zhijie Li, Jie Pu, Ting Yang, Yan Wang

**Affiliations:** ^1^ Department of Pharmacy, Affiliated Children’s Hospital of Jiangnan University (Wuxi Children’s Hospital), Wuxi, Jiangsu, China; ^2^ Department of Pharmacy, Affiliated Hospital of Jiangnan University, Wuxi, Jiangsu, China; ^3^ Center for ADR Monitoring of Jiangsu, Nanjing, Jiangsu, China; ^4^ Department of Dermatology, Affiliated Children’s Hospital of Jiangnan University (Wuxi Children’s Hospital), Wuxi, Jiangsu, China

**Keywords:** children, dupilumab, real world, safety, adverse drug reactions

## Abstract

**Objective:**

This study analyzes the occurrence and characteristics of adverse drug reactions (ADRs) of dupilumab in children in a real-world setting. It aims to enhance clinical practice and minimize medication safety risks in pediatric patients.

**Methods:**

This prospective study included children receiving dupilumab in the hospital between January 2022 and December 2023. Information on ADRs was collected and univariate and multivariate analyses were employed to identify high-risk factors for the occurrence of adverse effects in dupilumab treatment.

**Results:**

A total of 65 ADRs occurred in 1,103 treatments in 127 patients, with an incidence of 27.56% (35/127). A total of 62 patients aged 6 or below participated in this study, accounting for 48.82%. Univariate analysis showed that gender, age, duration of medication, frequency of dupilumab use were risk factors for the occurrence of adverse effects (*P* < 0.05). Multivariate logistic regression analysis showed that age [odds ratio [OR]: 0.071, 95% confidence interval [CI]: 0.012–0.433; *P* = 0.004] and frequency of dupilumab use (OR: 3.306, 95% CI: 1.078–10.135; *P* = 0.036) were risk factors for adverse effects. The outcomes of ADRs were improved in 10 cases (15.38%) and completely recovered in 55 cases (84.62%).

**Conclusion:**

Dupilumab has a good safety profile in Chinese children aged 6 months to 18 years for up to 2 years of treatment, with most adverse reactions being mild to moderate, and no serious ocular adverse reactions were reported. Age and frequency of dupilumab use were risk factors for adverse effects. Younger age and higher frequency of dupilumab use were associated with higher odds of ADRs.

## 1 Introduction

Dupilumab is a fully human monoclonal antibody derived from VelocImmune technology (1, 2) that targets the shared IL-4Rα subunit of IL-4 and IL-13 receptors, thereby inhibiting signaling pathways associated with these cytokines which are key drivers of type 2 inflammation (3–5). Dupilumab is approved in multiple countries, including Europe, the United States, and China, for treating moderate-to-severe atopic dermatitis in patients aged 6 months and older and asthma in those aged 6 years and older, chronic rhinosinusitis with nasal polyposis in adults, eosinophilic esophagitis in patients aged 1 year and older weighing at least 15 kg, prurigo nodularis in adults, and chronic obstructive pulmonary disease in adults (6–8). The safety of dupilumab has been demonstrated through clinical trials and post-marketing evaluations in adults and adolescents (9–17).

The common ADRs to dupilumab for atopic dermatitis treatment include (incidence ≥1%): injection site reactions, conjunctivitis, blepharitis, oral herpes, keratitis, eye pruritus, other herpes simplex virus infection, dry eye, and eosinophilia (6). However, safety data on children particularly Asian infants and those younger than 6 years of age undergoing long-term treatment remain limited (18, 19). Notably, there have been reports of serious adverse events such as uveitis following prolonged dupilumab use (20–22). Data from small cohorts and clinical trials (23–27) suggest that increased rates of dupilumab-related ocular surface disease in patients with atopic dermatitis are linked to more severe baseline disease, prior conjunctivitis, atopic comorbidities, eyelid involvement, elevated thymus and activation-regulated chemokine levels, serum IgE levels, and peripheral blood eosinophilia. However, prospective studies evaluating risk factors for dupilumab-associated ADRs in pediatric patients remain scarce. Real-world safety data on younger children are essential for clinicians to comprehensively assess the risk-benefit profile of dupilumab in China. This study aimed to evaluate the safety of dupilumab in Chinese pediatric patients in a real-world setting.

## 2 Materials and methods

### 2.1 Study design and eligible participants

A prospective, active monitoring study was conducted for pediatric patients who received dupilumab at least once between January 2022 and December 2023 at the Department of Dermatology, Affiliated Children's Hospital of Jiangnan University.

Inclusion criteria: (1) Children and adolescents aged 6 months to younger than 18 years; (2) Pediatric patients treated with dupilumab injection (300 mg/2.0 ml/vial or 200 mg/1.14 ml/vial pre-filled syringe, Sanofi Winthrop Industrie) administered subcutaneously.

Exclusion criteria: (1) Lack of informed consent from parents or legal guardians; (2) Patients with immunodeficiency, severe systemic diseases (e.g., cardiac, hepatic, renal), or malignancies; (3) Missing electronic medical records.

The study was approved by the Ethics Committee of the hospital (Ethics Approval No: WXCH2021-11-013) and adhered to the provisions of the Declaration of Helsinki. Standard pediatric clinical procedures and routine treatment protocols were followed by the attending physicians.

### 2.2 Data collection

Pediatric patient data were extracted from medical records, including demographic information (e.g., gender, age, weight), medication details (e.g., clinical diagnosis, administration time, dosage, route of administration, frequency, concomitant medications). The type, onset, management, and outcomes of ADRs were evaluated and recorded by the MSO (Medication Safety Officer) team during weekly follow-ups or when patients reported ADRs.

### 2.3 Criteria for adverse reaction evaluation

The causality assessment of all identified ADRs associated with dupilumab was performed using the WHO-Uppsala Monitoring Centre Criteria (WHO-UMC) (28). Two independent reviewers from the MSO team conducted the evaluations, with a third reviewer providing the final assessment in case of disagreement.

### 2.4 Statistical analysis

Data analysis was conducted using IBM SPSS version 26.0. Categorical variables were expressed as numbers (n) and percentages (%), normally distributed continuous variables as mean ± standard deviation (SD), and non-normally distributed continuous variables as medians and interquartile ranges [M (Q1, Q3)]. To identify risk factors associated with dupilumab-related ADRs, each candidate risk factor was initially analyzed using chi-square tests in a univariate model. Variables with statistical significance were then included in a multivariate logistic regression model. The dependent variable was the occurrence of ADRs (0 = No, 1 = Yes), and independent variables were analyzed to identify risk factors. Variables with a *P*-value < 0.05 in the multivariate logistic regression analysis were considered independent risk factors. A significance level of *P* < 0.05 was used.

## 3 Results

### 3.1 Analysis of baseline demographic characteristics and adverse reactions

A total of 127 pediatric patients met the inclusion criteria and were included in this study (Table 1). Of these, 84 (66.14%) were boys and 43 (33.86%) were girls. The age range of the patients was between 4.75 and 10 years, with 62 (48.82%) aged 6 years or younger. Nearly all patients (99.21%) were treated for moderate-to-severe atopic dermatitis, except for one patient treated for dystrophic epidermolysis bullosa. Of the 127 patients, 35 experienced ADRs, as shown in Table 1.

**Table 1:** Demographic characteristics of patients using dupilumab (n, %).

Baseline characteristics	Adverse reactions (*n*, %)	No adverse reactions (*n*, %)	Total (n, %)	*P*	*χ*²
Gender					
Male	28 (22.05)	56 (44.09)	84 (66.14)	**0**.042*^a^	4.143
Female	7 (5.51)	36 (28.35)	43 (33.86)		
Age					
6 months-3 years	5 (3.94)	2 (1.57)	7 (5.51)	**0**.025*^a^	5.005
3 years-18 years	30（23.62）	90（70.87）	120（94.49）		
Median (IQR)/years	7.20 (4.70–11.80)	7.00 (4.75–9.00)	7.00 (4.75–10.00)		
Weight					
<15 kg	3 (2.36)	7 (5.51)	10 (7.87)	0.586	1.927
≥15–<30 kg	17 (13.39)	56 (44.09)	73 (57.48)		
≥30–<60 kg	11（8.66）	21 (16.54)	32 (25.20)		
≥60 kg	4（3.15）	8 (6.30)	12 (9.45)		
Reason for medication					
Moderate to severe atopic dermatitis	35 (27.56)	91 (71.65)	126 (99.21)		
Nutritional bullous epidermolysis	0 (0.00)	1 (0.79)	1 (0.79)		
Duration of medication					
≤6 months	10 (7.87)	55 (43.31)	65 (51.18)	**0**.007**^a^	9.915
>6 months–≤12 months	13 (10.24)	20 (15.75)	33 (25.98)		
>12 months	12 (9.45)	17 (13.39)	29 (22.83)		
Median (IQR)/months	10.99 (4.64–17.41)	3.42 (0.29–9.15)	5.06 (1.23–11.48)		
Median (minimum-maximum)/months	10.99 (0.93–27.08)	3.42 (0.03–29.77)	5.06 (0.03–29.77)		
Frequency of dupilumab use					
≤10 times	17 (13.39)	75 (59.06)	92 (72.44)	**0**.000**^a^	18.901
≤20 times	10 (7.87)	13 (10.24)	23 (18.11)		
≤30 times	4 (3.15)	4 (3.15)	8 (6.30)		
≤40 times	4(3.15)	4(3.15)	4(3.15)		

^a^ Statistically significant findings.

* *P* < 0.05.

** *P* < 0.01.

To identify risk factors associated with dupilumab-related ADRs, five candidate risk factors were analyzed using chi-square tests in a univariate model, including gender, age, weight, duration of treatment, and frequency of dupilumab use. The results of the univariate analysis were presented in Table 1, and the results of the multivariate analysis were presented in Table 2. In the univariate model, variables that significantly influence the risk of ADRs included gender, age, duration of treatment, and frequency of dupilumab use (*P* < 0.05). In the multivariate logistic regression model, age [odds ratio [OR]: 0.071, 95% confidence interval [CI]: 0.012–0.433; *P* = 0.004] and frequency of dupilumab use (OR: 3.306, 95% CI: 1.078–10.135; *P* = 0.036) were identified as independent risk factors associated with dupilumab-related ADRs (*P* < 0.05, Table 2). Younger age was associated with increased ADRs risk (OR <1), while more frequent administration was also linked to a greater likelihood of ADRs (OR >1).

**Table 2:** Multivariate logistic regression analysis of risk factors for adverse reactions of dupilumab.

Variable	Coefficient	Standard error	Wald chi-square	*P*-value	OR	95% CI for OR
Gender	0.989	0.519	3.630	0.057	2.687	0.972–7.430
Age	**−2**.**642**^a^	0.921	8.229	**0**.004**^a^	**0**.**071**^a^	0.012–0.433
Duration of medication	0.78	0.577	1.827	0.176	2.181	0.704–6.755
Frequency of dupilumab use	**1**.**196**^a^	0.572	4.375	**0**.036*^a^	**3**.**306**^a^	1.078–10.135

^a^ Statistically significant findings.

* *P* < 0.05.

** *P* < 0.01.

### 3.2 Dupilumab treatment details

A total of 1,103 dupilumab treatments were administered to 127 patients (Table 3), with doses ranging from 200 to 600 mg via subcutaneous injection. Among these, 367 (33.27%) treatments involved off-label use, including off-age, off-dosage, off-frequency, and off-indication. There was no statistically difference in ADRs incidence between on-label and off-label use (*P* > 0.05) (Table 3). Then a detailed subgroup analysis of on-label vs. off-label use was conducted and the results were presented in Table 4. Similarly, no significant differences in ADRs incidence were observed between subgroups (*P* > 0.05). This suggests that off-label use of dupilumab in pediatric populations does not inherently increase the risk of ADRs compared to on-label use.

**Table 3:** Details of dupilumab administration (*n*, %).

Baseline characteristics	Adverse reactions (*n*, %)	No adverse reactions (*n*, %)	Total (*n*, %)	*P*	χ^2^
Off-label use					
Yes	15 (1.36)	352 (31.92)	367 (33.27)	0.290	1.118
No	41 (3.72)	695 (63.01)	736 (66.73)		
Dosage					
200 mg	9 (0.82)^a^	53 (4.81)	62 (5.62)	**0.001** ** ^b^	10.158
Higher than 200 mg	47 (4.26)	994 (90.12)	1,041 (94.38)		
Concomitant medications					
Yes	45 (4.08)	772 (69.99)	817 (74.07)	0.217	1.214
No	11 (1.00)	275 (24.93)	286 (25.93)		
Concomitant medications					
Monotherapy	11 (1.00)	275 (24.93)	286 (25.93)	0.339	5.235
TCI	36 (3.26)	561 (50.86)	597 (54.13)		
PDE-4 inhibitors	7（0.63)	86 (7.80)	93 (8.43)		
TCS + TCI	1 (0.09)	39 (3.54）	40 (3.63)		
TCS + PDE-4 inhibitors	0（0.00)	18 (1.63）	18 (1.63)		
TCS + Topical antibacterial agents	1(0.09)	68(6.17）	69(6.26)		

^a^ Compared with the group receiving a dosage higher than 200 mg.

^b^ Statistically significant findings.

** *P* < 0.01.

**Table 4:** Details of dupilumab off-label use (*n*, %).

Classification of dupilumab usage	Adverse reactions (*n*, %)	No adverse reactions (*n*, %)	Total (*n*, %)	*P*	χ²
Off-label age use	4 (0.36)	44 (3.99)	48 (4.35)	0.475	0.511
Within label age use	52 (4.71)	1,003 (90.93)	1,055 (95.65)		
Below recommended dosage	4 (0.36)	32 (2.90)	36 (3.26)	0.243	2.826
Within recommended dosage	41 (3.72)	793 (71.89)	834 (75.61)		
Above recommended dosage	11 (1.00)	222 (20.13)	233 (21.12)		
Within recommended frequency	52 (4.71)	892 (80.87)	944 (85.58)	0.112	2.529
Below recommended frequency	4 (0.36)	155 (14.05)	159 (14.42)		
Off-label indication	0 (0.00)	1 (0.09)	1 (0.09)		
Within label indication	56 (5.08)	1,046 (94.83)	1,102 (99.91)		

Concomitant medications were used in 74.07% of treatment regimens, with topical calcineurin inhibitors (TCI) being the most common combination. Other combination regimens included phosphodiesterase-4 (PDE 4) inhibitors, topical corticosteroids (TCS) combined with TCI or PDE-4 inhibitors, and TCS combined with antibacterial agents. No significant difference in ADRs incidence was found between combination therapy and monotherapy (*P* > 0.05) (Table 3). No significant differences in ADRs incidence were observed between subgroups (*P* > 0.05) (Table 3). In contrast, a significant difference in ADRs incidence was observed between the 200 mg and >200 mg dosage groups (*P* < 0.05).

### 3.3 Incidence of adverse reactions

Among the 1,103 treatments, 56 resulted in adverse reactions, totaling 65 ADRs (with some patients experiencing multiple ADRs in one treatment). The most affected systems were the respiratory system (22.05%), eyes (8.66%), skin and mucous membranes (7.09%), and the digestive system (5.51%). Nine ADRs were classified as severe, including herpes simplex virus infection, varicella infection, and prolonged abdominal pain and bloating (Table 5).

**Table 5:** Details of ADRs induced by dupilumab injection.

Affected organ/system	Clinical manifestation	Severity		Total cases (*n*)	Incidence (%)^a^	Cumulative incidence (%)^b^
		Mild (*n*)	Severe (*n*)			
Respiratory system	Upper respiratory tract infection	10	0	10	7.87	22.05
	Acute pharyngitis	3	0	3	2.36	
	Acute laryngitis	1	1	2	1.57	
	Acute tonsillitis	4	0	4	3.15	
	Acute bronchitis	7	0	7	5.51	
	Herpetic pharyngitis	1	0	1	0.79	
	Cough	1	0	1	0.79	
Eyes	Conjunctivitis	6	0	6	4.72	8.66
	Conjunctival hyperemia	2	0	2	1.57	
	Allergic conjunctivitis	2	0	2	1.57	
	Eye discomfort	1	0	1	0.79	
Skin and mucous membranes	Injection site redness and swelling	2	0	2	1.57	7.09
	Erythematous rash	3	0	3	2.36	
	Injection site pain	1	0	1	0.79	
	Injection site pruritus	1	0	1	0.79	
	Skin pruritus	1	0	1	0.79	
	Lip swelling	1	0	1	0.79	
Digestive system	Abdominal pain	2	2	4	3.15	5.51
	Abdominal bloating	0	1	1	0.79	
	Indigestion	1	0	1	0.79	
	Vomiting	1	0	1	0.79	
General disorders	Simple herpes	0	4	4	3.15	3.94
	Chickenpox	0	1	1	0.79	
Nasal area	Epistaxis	3	0	3	2.36	3.15
	Allergic rhinitis	1	0	1	0.79	
Nervous system	Dizziness	1	0	1	0.79	0.79

^a^ Incidence (%) = number of pediatric patients experiencing ADRs/total number of medication users × 100%.

^b^ Cumulative incidence (%) = cumulative number of pediatric patients experiencing ADRs/total number of medication users × 100%.

### 3.4 Occurrence and outcomes of ADRs

**Figure 1:**
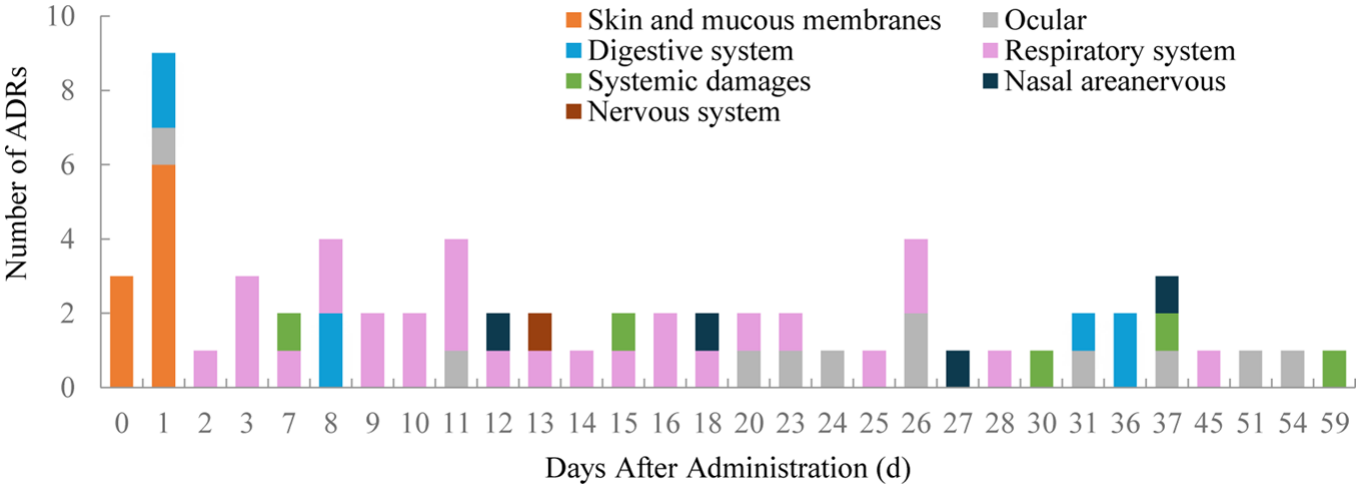
Distribution of time to occurrence of ADRs.

**Figure 2:**
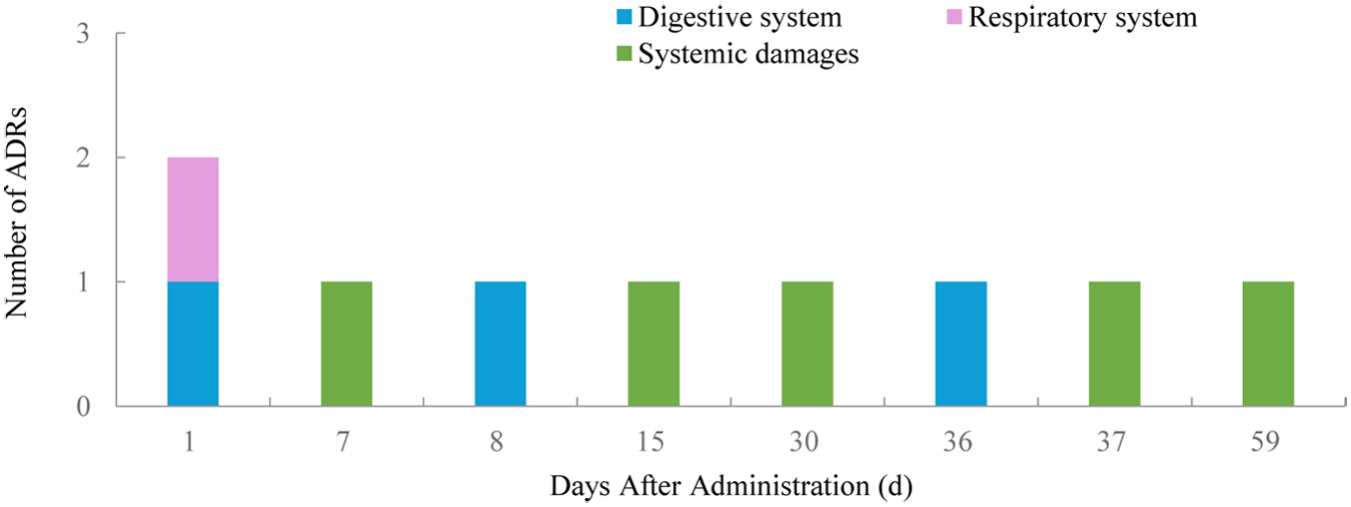
Distribution of time to occurrence of serious ADRs.

Of the 65 ADRs, 46.15% occurred within the first 11 days after the latest medication (Figure 1). During this period, most skin and mucosal injuries, respiratory diseases, and digestive reactions were observed. Ocular adverse reactions typically occurred 27.6 ± 15.6 days after the latest medication and 5.9 ± 4.6 months following the first dose. No cases led to treatment discontinuation. Distribution of time to occurrence of serious ADRs are presented in Figure 2. The occurrence time of severe ADRs was relatively scattered, with no central trend.

Among the ADRs outcomes, 10 cases (15.38%) showed improvement (including 2 cases of allergic conjunctivitis, 4 cases of conjunctivitis, 3 cases of epistaxis, and 1 case of allergic rhinitis), while 55 cases (84.62%) achieved total recovery. The ADRs in the eyes improved following the ophthalmologist's prescription of artificial tears, topical corticosteroid preparations, and topical anti-allergic treatments. The herpesvirus infection showed improvement after systemic or topical treatment with open-loop nucleoside antiviral drugs and topical administration of human interferon. The nasal ADRs were alleviated following treatment with vasoconstrictors, topical corticosteroids, and systemic anti-allergic agents. Improvements in respiratory system ADRs were observed after symptomatic treatment with inhaled corticosteroids, antibiotics, and mucus-diluting agents. In children presenting with unexplained abdominal pain, the therapeutic effects of probiotics, traditional Chinese patent medicines were not significant, and the abdominal pain ultimately resolved spontaneously.

### 3.5 Causality evaluation of adverse reactions

60 (92.31%) ADRs were deemed possible according to WHO-UMC criteria, as the events exhibited reasonable temporal relationships to drug intake; however, these ADRs could also be attributed to underlying diseases or other medications. 5 (7.69%) ADRs were deemed probable according to the WHO-UMC criteria. These events exhibited reasonable time relationships to drug intake, were unlikely to be associated with underlying diseases or other medications, and displayed clinically reasonable responses upon withdrawal. No definite ADRs were identified.

## 4 Discussion

Our study is among the initial studies to provide prospective real-world evaluations of the safety of dupilumab in Chinese pediatric patients. The findings demonstrate that dupilumab is generally safe for use in children aged 6 months to 18 years, with most ADRs being mild to moderate in severity. Importantly, no serious ocular adverse reactions were reported, which aligns with previously documented safety profiles from other international studies (11–16, 29–32). However, our data underscore the need for vigilance regarding specific patient subgroups that may be at higher risk for ADRs.

The incidence of ADRs in our cohort (27.56%) falls within the range of those observed in other safety studies involving pediatric populations receiving dupilumab (33). ADRs mainly involves the respiratory system, eyes, digestive system, skin and mucous membranes, systemic damage, nose, and nervous system. A systematic review and meta-analysis (34) on the efficacy and safety of dupilumab in children and adolescents with atopic dermatitis, which included 18 studies conducted in Europe, North America, and Asia, reported that most ADRs were mild to moderate and manageable. Common adverse events included exacerbation of atopic dermatitis (12.9%), injection-site reactions (7.3%), nasopharyngitis (10.6%), upper respiratory tract infections (10.1%), headache (7.5%), conjunctivitis (6.6%), eosinophilia (43.5%), and herpes viral infections (7.9%). Although potential biases related to institutional factors existed, the types of ADRs and the incidence of ADRs observed in our study were largely consistent with those reported in the systematic review and meta-analysis. However, there were some differences, for instance, eosinophilia was not observed in our study, as routine blood testing was not conducted during standard treatment in our hospital.

Respiratory related ADRs were the most frequently observed adverse reactions in this study (22.05%), which aligns with findings from other safety studies on dupilumab (35–39). Further investigation of its association with dupilumab is warranted.

Other common ADRs associated with dupilumab included ocular reactions (8.66%), skin and mucous membrane reactions (7.09%), and digestive system reactions (5.51%). Ocular adverse reactions typically occurred 27.6 ± 15.6 days after the latest medication and 5.9 ± 4.6 months following the first dose, consistent with previous reports (27, 40, 41). A Phase III randomized, double-blind, placebo-controlled trial reported similar rates of ocular adverse reactions in patients aged 6 months to under 6 years with uncontrolled atopic dermatitis: allergic conjunctivitis (1%), conjunctivitis (4%), and stenosing conjunctivitis (5%) (11). A multicenter Canadian case series involving pediatric patients under 12 years of age with moderate-to-severe atopic dermatitis was conducted across six Canadian sites. This study reported that conjunctivitis was diagnosed in 8 out of 96 patients (8.3%), with the first episode occurring on average after 10.5 ± 7.71 weeks. No serious adverse events were recorded, and none of the children discontinued treatment with dupilumab (42). Our findings are largely consistent; however, no cases of stenosing conjunctivitis were observed. At the next follow-up, all ocular reactions had either resolved or were in the process of resolution. The team referred six cases of conjunctivitis and two of allergic conjunctivitis to ophthalmology and showed improvement after treatment with artificial tears, topical corticosteroids, and antiallergic agents. Other studies have noted severe ocular reactions, such as uveitis, occurring 7 months to 2 years post-treatment (39), indicating the need for vigilance regarding delayed-onset ocular ADRs in long-term dupilumab users. The exact pathophysiological mechanisms are not fully understood (43). Some studies indicate that monoclonal antibodies, by binding to IL-4Ra and inhibiting IL-4 and IL-13, can prevent the activation of conjunctival goblet cells. This inhibition results in hypoplasia and a reduction in mucin production, which subsequently affects the stability of the tear film and the functionality of the mucosal epithelial barrier (44, 45). In our study, the longest duration of dupilumab use was 29.8 months, with a median duration of 5.06 months (range 1.23–11.48 months), and no severe ocular ADRs were observed. But this may be attributed to the insufficient follow-up duration. Therefore, extended observational studies are essential for evaluating the long-term safety of dupilumab, especially concerning ocular and systemic effects that may arise with prolonged use. A recent study has demonstrated that the patient's age, history of conjunctivitis, and higher baseline Eczema Area and Severity Index (EASI) score are significantly associated with dupilumab-associated ocular surface disease (46). Previous studies also indicate that the risk of dupilumab-related conjunctivitis is associated with early-onset atopic dermatitis (AD) and eosinophilia (>500/mm^3^) (11), and that the prophylactic use of artificial tears may mitigate this risk (47, 48). Clinical vigilance is recommended, and some studies suggest restricting dupilumab prescriptions in patients with severe ocular diseases due to known adverse events (29).

Digestive related ADRs, involving abdominal pain and bloating, were consistent with previous reports (11, 33). By blocking the activity of IL-4/IL-13 receptors, dupilumab may potentially lead to parasitic infections for users because both cytokines contribute to the elimination of parasites through increased mucus production and eosinophilic mucosal inflammation in the gut (35, 49–52). Thus, if patients present with unexplained abdominal pain, diarrhea, nausea, vomiting, or severe nocturnal perianal pruritus, examination for parasites in stool or the perianal area is advised. In addition, Yosuke Shimodaira et al. described the case of a 17-year-old Asian male patient with severe AD who had a history of pediatric asthma and attention-deficit hyperactivity disorder and experienced the occurrence of intermittent abdominal pain, tenesmus, and diarrhea seven times a day after 3 months of dupilumab treatment, subsequently being diagnosed with ulcerative colitis (53). In our study, two cases of abdominal pain were reported, lasting 26 and 10 days, respectively, presenting as paroxysmal dull pain around the umbilicus, without vomiting and with slightly loose stools. The helicobacter pylori test yielded negative results, and the stool routine was normal. An abdominal ultrasound, along with examinations of the liver, gallbladder, spleen, and pancreas, revealed normal findings. Furthermore, fecal examinations were unremarkable, with no parasites detected around the anus. The treatment effects of probiotics and traditional Chinese patent medicines were not significant. These results may be attributed to the local gastrointestinal exposure of the drugs and their limited systemic absorption; however, the specific mechanisms underlying these effects remain unclear and warrant further investigation.

Nine cases of severe ADRs were observed, including abdominal pain, bloating, and infections caused by herpes simplex and varicella, which warrant particular attention. Although the majority of these severe ADRs resolved with appropriate treatment, it is crucial to educate both caregivers and clinicians about the potential for infectious complications, especially when treating children who may have additional risk factors for infection.

Notably, no significant difference was found in ADRs incidence between on-label and off-label uses of dupilumab. In our study, off-label use includes off-age use, off-dosage use, off-frequency use, and off-indication use. For off-age use, dupilumab was administered to children aged 2 years and 11 months to 6 years between January 1, 2022, and May 30, 2023 when dupilumab was only approved for children aged 6 years old and above in China. Off-dosage use includes two types: below the recommended dosage and above the recommended dosage. The doctor's evaluation of the specific condition of the child and the actual availability of drug specifications were common reasons for these deviations. Off-frequency use often involved extending dosing intervals during maintenance therapy for atopic dermatitis due to the high cost of dupilumab. Although off-label use of dupilumab in pediatric populations does not inherently increase the risk of ADRs compared to on-label use, it remains essential to evaluate each case individually, especially given the complex nature of off-label treatment and the lack of long-term data for such uses.

Our multivariate analysis identified younger age and frequency of dupilumab use as independent risk factors for ADRs. Younger age was associated with higher odds of ADRs. This is consistent with the known pharmacokinetic and pharmacodynamic differences in younger children, whose developing organ systems may lead to altered drug metabolism and increased vulnerability to adverse reactions (54, 55). Based on this finding, here are some recommendations for managing the use of dupilumab in pediatric populations. Healthcare providers should monitor intensively during the initial months of therapy for younger children (≤3 years) to detect ADRs early and focus on mild to moderate ADRs because they are common. In addition, healthcare providers should educate caregivers and parents on recognizing early symptoms such as rash, conjunctivitis, or local injection site reactions, given that young children may have difficulty expressing discomfort or other symptoms (56). Healthcare providers could provide symptomatic management and supportive care for mild reactions. For moderate ADRs, consider temporary dose adjustments or adjunctive treatments. At the same time, monitoring for rare or delayed onset ADRs should still be part of follow-up plans, especially for high-risk groups. And establish protocols for immediate referral to specialists (e.g., dermatologists, ophthalmologists) if severe reactions occur. Caregivers should be able to recognize ADRs such as skin rashes, redness, and mild swelling at the injection site, eye-related symptoms like redness or mild irritation and notify healthcare providers if symptoms persist, worsen, or if new symptoms (e.g., fever or severe rash) occur. It's very important for caregivers to document and report all side effects to the healthcare provider, even if they appear mild. As younger children have a higher likelihood of experiencing ADRs, caregivers should be vigilant in observing their reactions and symptoms after each treatment. Frequent use of dupilumab was another factor that associated with an increased risk of ADRs in this study. The association between frequent dupilumab use and ADRs suggests that cumulative exposure may increase the likelihood of immune modulation leading to adverse events. Clinicians should monito regularly to detect early signs of ADRs, particularly in high-risk organ systems such as the skin, respiratory tract, and gastrointestinal system and consider dose intervals and cumulative exposure when tailoring treatment regimens, particularly in younger or more vulnerable pediatric patients.

In addition, the identified risk factors emphasize the need for tailored treatment approaches in pediatric populations. Developing risk stratification tools that incorporate age, frequency of use, and other potential factors-such as nutritional status, baseline disease severity, and genetic predispositions can help clinicians predict and mitigate ADRs risks. These findings also highlight the need for longitudinal studies to explore long-term safety profiles and late-onset ADRs, particularly in younger age groups. Investigating genetic, environmental, and cultural factors that may influence susceptibility to ADRs could further enhance the understanding of dupilumab's safety. And collaborative multi-center studies involving diverse patient populations are necessary to validating these findings and developing universal clinical guidelines.

This study has several limitations. In one hand, it was conducted at a single tertiary care institution in China, and the findings may not fully represent broader populations or other healthcare settings. The geographic location of the study could introduce biases due to differences in environmental factors, regional disease prevalence, and healthcare access. The clinical protocols and prescribing practices at our center may not reflect those in other healthcare settings, potentially influencing the observed safety profile of dupilumab. Moreover, cultural factors, such as caregiver reporting tendencies and treatment adherence, may vary across regions and countries. While our study provides valuable real-world safety data, future multi-center studies involving diverse geographic locations are essential to validate these findings and provide a more comprehensive understanding of dupilumab's safety profile in pediatric populations. In the other hand, the maximum follow-up period of 2 years may not capture long-term ADRs or late-onset effects, particularly ocular adverse events like uveitis, reported in other studies. Future studies should extend follow-up duration to evaluate long-term safety, especially for ocular and systemic effects. Additionally, our sample size, while adequate for identifying common ADRs, may not be sufficient to capture rare adverse events.

## 5 Conclusion

In conclusion, our real-life data demonstrated that dupilumab has a good safety profile in Chinese children aged 6 months to 18 years for up to 2 years of treatment, with most adverse reactions being mild to moderate, and no severe ocular reactions were observed. The safety data were consistent with the known safety profile of dupilumab. Age and frequency of dupilumab use were identified as risk factors for the occurrence of adverse effects. Therefore, adverse effects should be closely monitored in patients with these risk factors.

## Data Availability

The original contributions presented in the study are included in the article/Supplementary Material, further inquiries can be directed to the corresponding authors.
